# Remarks Shewing the Expediency of Some Legislative Enactment to Insure Competent Medical and Surgical Aid to Sick and Hurt Poor

**Published:** 1817-11

**Authors:** John C. Yeatman


*For the London Medical and Physical Journal.*

### *Remarks shewing the expediency of some Legislative Enactment to insure competent Medical and Surgical Aid to Sick and Hurt Poor;* by JOHN C. YEATMAN, Esq.

——— Miseris succurrere disco.

THE Faculty is indebted to the Worcestershire Medical and Surgical Society for their intended Bill, as far as it regards the *casual* poor; and it is to be hoped that the "Associated Apothecaries of England and Wales" will consult the interest of the poor and of themselves by petitioning Parliament to make a better provision than the one existing for medical attendance upon paupers *generally*. And the Bill for the better regulation of the Poor-laws, begun in the last session with so much wisdom, certainly affords the best opportunity for an address of the foregoing kind. Concerning the casual poor, it were a crying evil and a shame if "the present system of removing paupers on account of application, in cases of illness, to the overseers of the parish in which they happen to reside, to that parish to which they belong," *be continued*, when "it often deprives the poor family of the means of gaining a living, and frequently induces them not to apply for a suspended order; while, if a medical man is called in, under such circumstances, to attend them, he has no legal means of obtaining any remuneration for his attendance."*

---

* See the Report of the Resolutions of the Worcestershire Medical and Surgical Society in the Lond. Med. and Phys. Journal for July, p. 79.

In

In reference to a situation in which the casual poor and their medical attendants are every day placed, I would remark, that, although the law, I believe, compels the parish in which a pauper meets with such an accident that he cannot be removed, to provide him with surgical assistance, obliging that to which he belongs to repay the necessary expences incurred; yet in a majority of cases the charge for attendance, &c. passes without notice, overseers shrewdly and meanly suspecting that a practitioner would rather lose it than incur the enmity of the vestrymen or principal parish-paymasters, by seeking his remedy in a court of justice. Hence, except in certain cases of dreadful accident, in which the expence incurred for distant visits and much medicine has determined him on seeking redress from an English jury, he is ever yielding to the lesser evil of the two, by submitting to heavy losses, as it respects time, labour, medicine, and surgical applications; till, at length, being unable or unwilling to sacrifice so much, *he is induced to withdraw assistance from the sick and hurt poor to a very great extent.* Of more than two thousand cases attended by me (as appears by my Register), while engaged in the parish surgeoncy of Frome, for one year ending March last, two hundred and sixty persons, *I know*, belonged to other parishes; and, to those who are acquainted with country practice, I need not add, that, having requested remuneration in a few of the most serious cases, it has been, as usual, unattended to. In answer to this, it may be urged, that a written order from the overseer to attend each sick or hurt pauper should be obtained before assistance is given; but, to say nothing of the occasional danger to the pauper in the interval between the application and supposed receipt of an answer, that order is in vain solicited both by the friends of the pauper and by a note from the surgeon, specifying the nature of the case; while, if it be known to the overseer that professional aid has been given, and is continued, it is almost always refused, with the pitiful view to elude payment.

But, to advert to a most essential benefit to be obtained for paupers *generally*, and for the credit of the profession: it appears to me that a Bill, besides containing the spirit of the two resolutions of the Worcestershire Medical and Surgical Society, should go on to implore Parliament to put down that *impolitic* and *cruel* practice of FARMING OUT the medical and surgical care of parishes to the *lowest bid*[illegible], whereby the health of paupers is every hour compromised by a system which, in its operation, directly leads to neglect, an inadequate supply of medicine, and to unskilful treatment.

Country

Country practitioners should be particularly alive to this subject.

To point out an adequate remedy for the numerous evils arising out of the present system of supplying medical and surgical aid to sick and hurt paupers, were an honour worthy of the greatest philanthropist!

*Frome; Oct.* 12, 1817.

